# Self-Healing Mechanism and Conductivity of the Hydrogel Flexible Sensors: A Review

**DOI:** 10.3390/gels7040216

**Published:** 2021-11-16

**Authors:** Juan Zhang, Yanen Wang, Qinghua Wei, Yanmei Wang, Mingju Lei, Mingyang Li, Dinghao Li, Longyu Zhang, Yu Wu

**Affiliations:** 1 Industry Engineering Department, School of Mechanical Engineering, Northwestern Polytechnical University, Xi’an 710072, China; juanzhang@mail.nwpu.edu.cn (J.Z.); 2019100156@mail.nwpu.edu.cn (Y.W.); leimj@mail.nwpu.edu.cn (M.L.); limingy@mail.nwpu.edu.cn (M.L.); lidinghao@mail.nwpu.edu.cn (D.L.); longyu@mail.nwpu.edu.cn (L.Z.); wuyu@mail.nwpu.edu.cn (Y.W.); 2 Institute of Medical Research, Northwestern Polytechnical University, Xi’an 710072, China

**Keywords:** flexible sensor, self-healing mechanism, conductivity, mechanical lifetime, hydrogel

## Abstract

Sensors are devices that can capture changes in environmental parameters and convert them into electrical signals to output, which are widely used in all aspects of life. Flexible sensors, sensors made of flexible materials, not only overcome the limitations of the environment on detection devices but also expand the application of sensors in human health and biomedicine. Conductivity and flexibility are the most important parameters for flexible sensors, and hydrogels are currently considered to be an ideal matrix material due to their excellent flexibility and biocompatibility. In particular, compared with flexible sensors based on elastomers with a high modulus, the hydrogel sensor has better stretchability and can be tightly attached to the surface of objects. However, for hydrogel sensors, a poor mechanical lifetime is always an issue. To address this challenge, a self-healing hydrogel has been proposed. Currently, a large number of studies on the self-healing property have been performed, and numerous exciting results have been obtained, but there are few detailed reviews focusing on the self-healing mechanism and conductivity of hydrogel flexible sensors. This paper presents an overview of self-healing hydrogel flexible sensors, focusing on their self-healing mechanism and conductivity. Moreover, the advantages and disadvantages of different types of sensors have been summarized and discussed. Finally, the key issues and challenges for self-healing flexible sensors are also identified and discussed along with recommendations for the future.

## 1. Introduction

In recent years, research on flexible sensors in the fields of wearable devices [1,2,3], health monitoring [4,5,6,7,8], and electronic skin [9,10,11] and smart sensor systems [12,13,14] has attracted widespread attention. Due to its good flexibility and ductility, high sensitivity and small size, the flexible sensor can better monitor subtle changes in the external environment, such as temperature [15,16,17], humidity [18,19], pressure [20] and deformation [21,22]. Compared with traditional sensors based on rigid semiconductors, metals and ceramics, flexible sensors can convert subtle changes into detectable electric signals. However, the accuracy and stability of the signal transmitted by the flexible sensor are closely related to the sensor’s external environment and service lifetime [23]. To adapt to harsh working environments, self-healing flexible sensors have been proposed in different fields. According to the matrix material composition, self-healing flexible sensors can be divided into self-healing flexible elastomer sensors [24,25,26,27,28,29,30,31,32,33] and self-healing flexible hydrogel sensors [34,35,36,37,38,39,40,41,42]. 

Flexible elastomer sensors usually use flexible metals [43,44], polymer films [45,46] and polymer elastomers [47,48] as substrates and are then combined with graphene [49,50], carbon [51,52], carbon nanotubes (CNTs) [53,54] and metal nanowires [55,56] as the conductive fillers to form the final sensors. However, due to the inherent properties of flexible metals, polymer films and elastomers, these sensors always exhibit limited stretchability and poor fatigue resistance. Conversely, the self-healing flexible hydrogel sensor has excellent flexibility and biocompatibility, and simplicity in preparation, which has attracted the attention of many researchers [57,58,59,60].

Improving the mechanical properties (e.g., tensile strength, compressive strength, elongation at break and fatigue resistance) and conductivity are the keys to preparing self-healing flexible hydrogel sensors. Mechanical properties are an essential requirement for sensors and determine their working environment and lifetime. Conductivity is another important element for sensors and directly affects the sensitivity and stability of the sensor [61]. However, the commonly used hydrogels often have deficiencies in both mechanical properties and conductivity. To improve the performance mentioned above, one common method is incorporating another component into the hydrogel matrix, such as conductive polymers or nanoparticles [62,63], to improve the mechanical properties and conductivity of the hydrogel. These introduced components can form a conductive network in the hydrogel to transfer electrons and finally result in good conductivity [64]. Generally, the higher the content of the filling components, the better the conductivity, while more filling components in hydrogels easily promote filler agglomeration and result in phase separation between the filling component and polymer matrix, subsequently reducing the stretchability, toughness, fatigue resistance and self-healing property of the sensors [65,66,67,68]. Typically, surface modification of the conductive filler or cross-linking agent is used to improve the affinity of the conductive filler to the hydrogel matrix and inhibit phase separation. Another form of conductivity of hydrogels is ionic transmission [69]. For ionic conductive hydrogels, adding salts (e.g., LiCl, NaCl or KCl) to the hydrogel network creates a large number of freely moving ions. Thus, ionic hydrogels can enhance conductivity while maintaining good mechanical properties [70,71]. Furthermore, ionic hydrogels have better transparency than electronic conductive hydrogels, allowing their applications in the biomedical field [72,73].

**Figure 1:**
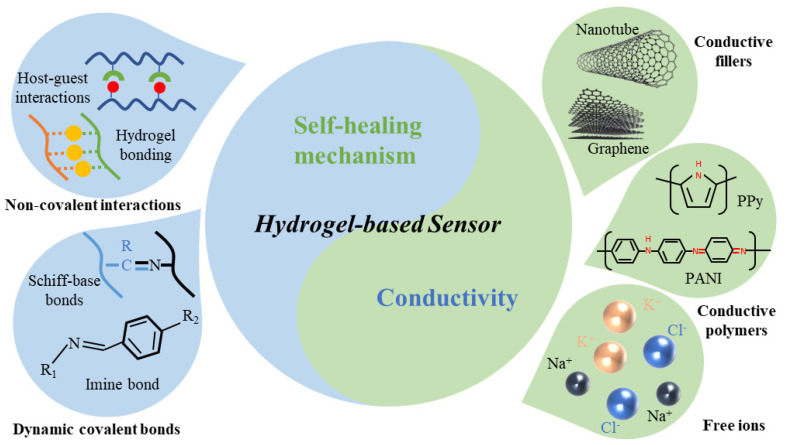
Illustration of self-healing mechanism and conductivity of hydrogel-based sensor.

Over the past few decades, self-healing hydrogels have been extensively investigated due to their conductivity and self-healing properties, and many outstanding contributions have been obtained. However, few review articles focusing on the self-healing mechanism and conductivity of hydrogel sensors have been reported, and almost no article can give us a comprehensive understanding of the mechanism and conductivity of self-healing hydrogel sensors. Many recent review articles mainly focus on the fabrication and application of hydrogel sensors [74,75,76,77,78]. The goal of this review is to provide researchers with a systematic and comprehensive understanding of the self-healing mechanism and conductivity of flexible hydrogel sensors. As summarized in Figure 1, the route to achieve self-healing of hydrogels is by noncovalent or reversible dynamic covalent bonding in polymeric materials, and enhancing the conductivity of hydrogels by the addition of conductive fillers, conductive polymers or conductive ions. In addition, we aim to help researchers design and manufacture flexible sensors according to the self-healing mechanisms and conductive categories.

This review aims to provide a comprehensive account of the latest progress in self-healing flexible hydrogel sensors. First, we summarize the mechanism of self-healing flexible materials and their latest developments in flexible sensor applications. Second, the conductive categories of the self-healing hydrogel flexible sensor were reviewed. This study ends with a brief conclusion and perspective on this rapidly developing and promising field of flexible sensors.

## 2. Self-Healing Mechanism of Hydrogel

In recent years, breakthroughs have been made in research on hydrogels, but most hydrogels still have poor mechanical strength and are susceptible to damage (accidental fracture, etc.), leading to some microscopic or macroscopic cracks [79,80]. As these cracks are further extended, the structure of the hydrogel network is destroyed, its mechanical properties are significantly reduced and its original function is lost, resulting in a waste of resources. To reduce environmental pollution and save resources, it is necessary to study self-healing materials that can prolong life cycles via the autonomous repair of damage [81]. The self-healing ability allows the hydrogel to recover from the damage it has sustained, thus maintaining its main properties and functions, and finally extending the service lifetimes of the materials [82,83,84]. The self-healing properties of polymeric materials can be divided into extrinsic and intrinsic self-healing, depending on whether the self-healing component is inserted into the polymer or the original component in the polymer matrix. Extrinsic self-healing materials can heal by encapsulating the components that enable healing, such as monomers, that are dispersed in matrix materials in the form of capsules, and the components inside the capsules are released upon damage. This method has difficulty achieving repeated self-healing. In the second category of intrinsic self-healing materials, healing is achieved through noncovalent or reversible dynamic covalent bonds in polymeric materials. When a hydrogel is subjected to external forces, the covalent or noncovalent bonds in the gel will break, forming a fracture surface. By re-contacting the fracture surface, the polymer chain segments interpenetrate and re-establish the dynamic cross-linking sites in the damaged area to repair the network structure of the hydrogel and restore its original mechanical properties and function to a certain extent. Table 1 compares the different performance (such as self-healing time, efficiency and mechanical property recovery) of different hydrogels in detail.

### 2.1. Noncovalent Interactions

Noncovalently cross-linked hydrogels have been developed to assemble self-healing hydrogels using various mechanisms, including hydrophobic interactions, hydrogen bonding, host–guest interactions and metal coordination leading to dynamic and reversible networks. When the hydrogels are subjected to an external force, the noncovalent interaction in the network will dissociate and associate, and the hydrogel will have hysteresis in the process of deformation and recovery, dispersing the energy [98]. Thus, the hydrogels exhibit reproducible features and a fascinating self-healing ability. However, these hydrogels have stimuli responses and are less mechanically robust structures.

#### 2.1.1. Hydrophobic Associations

Hydrophobic associated hydrogels are physically cross-linked hydrogels formed by hydrophobic interactions [85]. The preparation of hydrophobic association hydrogels generally adopts the micellar polymerization method [99]. Micelle polymerization is formed by introducing the hydrophobic segment into the hydrophilic polymer segment for copolymerization, and the hydrophobic segment serves as the dynamic cross-linking point of the hydrogel. When the hydrogel is stretched, these physically cross-linked points could dynamically dissociate/associate to reorganize the polymer chains, distributing the applied stress uniformly over the entire network. Meanwhile, physically cross-linked points dissipate the energy by a large hysteresis [86]. In micellar polymerization, hydrophilic segments, hydrophobic segments and surfactants are required [87,88]. Tuncaboylu et al. [100] reported a hydrophobic interaction self-healing hydrogel. Using stearyl methacrylate as the hydrophobic monomer and n-alkyl(meth)acrylate as the physical cross-linking agent, copolymerization in wormlike sodium dodecyl sulfate (SDS)/NaCl aqueous solutions was performed to prepare the hydrogel. Additionally, the effects of the length of the alkyl side chain of the hydrophobe and the surfactant concentration on the properties of the self-healing gel are discussed.

**Figure 2:**
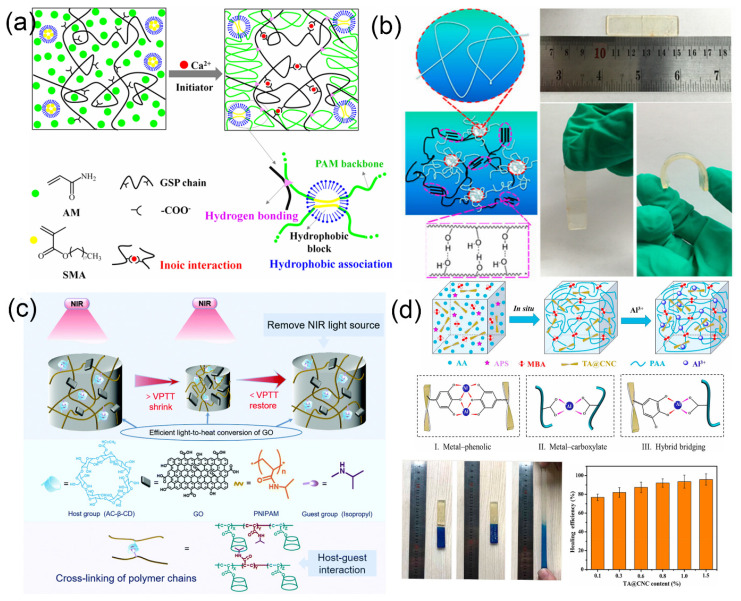
Physically cross-linked hydrogels with a different type of noncovalent interactions. (**a**) The GSP-HPAM composite hydrogel with hydrophobic association; (**b**) schematic illustration for PVA/CP hydrogel, and self-healed by hydrogen bonding; (**c**) schematic diagram of internal structure of PNIPAM-b-CD/GO hydrogel based on host–guest interaction; (**d**) schematic illustration of hydrogel synthetic process, coordination modes and self-healing properties.

To enhance the mechanical properties of hydrogels, self-healing hydrogels are usually designed by combining hydrophobic association effects with other physical interactions [101]. A composite hydrogel was prepared by incorporating grape seed-extracted polymer (GSP) into an acrylamide, methacrylate stearate matrix [102]. As the side chains of GSP contain carboxyl groups, ammonia groups, hydroxyl groups and alkyl groups, these groups tend to form dynamic noncovalent bonds (hydrogen bonds, ionic interactions and hydrophobic association) in the hydrogel, which could dissipate energy efficiently. The hydrophobic association existing in the system can self-heal after being broken, which gives the hydrogel excellent mechanical properties and self-healing properties, as shown in Figure 2a.

Yang et al. [103] proposed a polyacrylamide (PAM)/cellulose nanofiber (CNF)/multiwalled carbon nanotube (MWCNT) hydrogel by in situ polymerization. CNFs dispersants uniformly disperse the MWCNTs in the hydrogel and strengthen the mechanical properties of the hydrogel by hydrophobic interactions and electrostatic repulsion. The prepared hydrogel had conductivity, an electromagnetic shielding function and self-healing properties. The hydrogel could be bent 1000 times without breaking after self-healing. The hydrogel could completely self-heal in approximately 7 days, with a healing efficiency of 77.2%.

#### 2.1.2. Hydrogen Bond

Hydrogen bonding, as a type of physical interaction, is formed by the short-range supramolecular interaction between an electron-deficient hydrogen atom and an electron-rich species [89,104]. The hydrogen bond can be broken by heating. It can also be regenerated at a certain temperature. This reversible effect enables the material to achieve self-healing effects [90]. Due to the inherent weakness of hydrogen bonding, hydrogen bonding can be susceptible to competition with the surrounding water molecules, potentially weakening the mechanical properties of hydrogels. 

Improvement in the mechanical properties of hydrogels can be achieved by designing the network structure of the hydrogel, such as a double-network hydrogel. A double-network hydrogel with poly (acrylamide-co-acrylic acid) (PAM-co-PAA) as the first network and polyvinyl alcohol (PVA) as the second network was prepared [105]. The first network was formed by free radical copolymerization, and the second network was created by freezing and thawing a large number of hydrogen bonds as cross-linking points. The mechanical properties and self-healing properties of the hydrogel were improved by these hydrogen bonds as shown in Figure 2b. The hydrogen bonds can also be derived from the interaction of C=O and N-H, in addition to the hydroxyl groups. 

In addition, hydrogen bonding is often combined with other cross-linking interactions to produce self-healing hydrogels with excellent mechanical strength. The self-healing hydrogel was also prepared by carboxymethyl cellulose (CMC) in a paste with water and acidified with a citric acid solution [106]. The self-healing effect was the best when the hydrogel was soaked in citric acid at a concentration of 8 mol/L. When the hydrogel was cut in half and re-contacted, the uncross-linked CMC built new hydrogen bonds with hydrogen ions, thereby restoring the damaged area of the hydrogel. The self-healing efficiency reached 81%, and the compressive strength reached 2.3 MPa. Hydrogen bonds can also work with other chemical bonds to improve the mechanical properties of the hydrogel. Wang et al. [107] added acrylic acid and methylene bisacrylamide to a mixed solution of cellulose and PVA, and a double-network hydrogel was obtained by UV-induced polymerization. The cutting hydrogel contacted for 16 h, and the cracks disappeared completely and could be bent, at room temperature. This double-network structure improved the mechanical properties of the hydrogel. In addition, the self-healing properties of the hydrogel were also improved by forming hydrogen bonds and metal coordination bonds.

In addition, the introduction of 2-uridine 4-pyrimidinone (UPy) in the preparation of hydrogels has enabled excellent self-healing properties. UPy has been widely used as a multi-hydrogen bonding motif in supramolecular chemistry due to its higher intermolecular bonding strength than single hydrogen bonds. For example, the UPy group was used as a cross-linking point with a PANI/PSS network to form a self-healing conductive hydrogel [108]. The hydrogel completely self-heals within 30 s after damage due to the multiple hydrogen bonds generated by UPy. Furthermore, the combined effect of multiple hydrogen bonds and metal–ligand coordination not only enables the hydrogel to achieve rapid self-healing, but also improves the mechanical properties of the hydrogel (the tensile strength of the self-healed hydrogel reached 7.9 MPa). This hydrogel also has excellent self-healing properties. The damaged hydrogels can recover 91% of their initial properties within 1 h [109]. 

#### 2.1.3. Host–Guest Interaction

The host–guest interaction is a type of noncovalent interaction formed by the physical insertion of the guest’s moiety into the host moiety [91]. Generally, host molecules include cyclodextrins (CDs), pillar[n]arenes, crown ethers, calix[n]arenes, cucurbiturils and adamantane. The commonly used guest molecules include ferrocene, azobenzene, cholic acid and N-vinylimidazole derivatives.

Among the frequently used host molecules, CD has lipophilic inner cavities and hydrophilic outer surfaces, enabling high-affinity interactions with specific hydrophobic guest moieties. Specifically, as the most important member of the CD family, β-cyclodextrin (β-CD) is most widely produced and possesses a cavity that matches the size of numerous guest molecules and can be easily crystallized, separated and purified. Furthermore, β-CD inclusion complexes can enhance the resistance of the encapsulated guest molecules to various environments, such as acidic and alkaline media, light and heat [110,111,112]. A self-healing hydrogel was synthesized by the host–guest interaction between the hydrophobic isopropyl group of N-isopropylacrylamide (NIPAM) and β-CD [110]. The main procedure is shown in Figure 2c. The isopropyl group in NIPAM serves as the guest component and β-CD serves as the host component to form a host–guest complex. Hydrogels have a variety of hydrogen bonds and host–guest interactions. Extensive comparative experiments have shown that the host–guest interaction is the principal factor influencing the self-healing of hydrogels. The hydrogel, cut into two pieces, is capable of rapid self-healing at room temperature. The self-healing ability of the hydrogel was measured by its weight-bearing capacity before and after healing. For example, the original hydrogel (before cutting) could bear 200 g and after healing it could bear 55 g. Therefore, the self-healing efficiency is approximately 28%. Adamantane, as the guest molecule, can form a stable inclusion complex with the β-CD cavity and has a high binding constant with the β-CD cavity compared with other guest molecules. Rodell et al. [113] used methacrylate to modify hyaluronic acid and further used it as the main chain of β-cyclodextrin/amantadine (β-CD/Ad) to prepare a double network hydrogel with self-healing properties. The cross-linking point of the first network was formed by the host and guest interaction, and the second network was a methacrylate network. Not only were the mechanical properties greatly improved, but self-healing could also be completed in an instant. The experimental results show that the cut hydrogel fragments heal quickly within about 1 s. However, hydrogels synthesized by chemical processes take a long time and produce toxic byproducts that are unsuitable for biological applications. Therefore, a nonchemical grafting method to prepare hydrogels was proposed [114]. In this hydrogel, the amphiphilic substance N,N-dimethyl-1-adamantane (DM-AD) was used as a cross-linking agent, and CMC and poly β-cyclodextrin (β-CDP) were used as the polysaccharide skeleton. One end of DM-AD is the adamantly group, which is wrapped by β-CDP through host–guest interactions. The nitrogen atom at the other end combines with protons to form a quaternary ammonium compound and is electrically attracted to the carboxyl anion. To verify the self-healing ability of the hydrogel, two identical hydrogels were stained with different colors and cut from the middle of hydrogel. Take two different colored hydrogel cut surfaces into contact with each other. After much time (more than 0.5 h), the hydrogel was completely healed, and there was no obvious sign of fracture on the fracture surface of the hydrogel. In addition, stretching the ends of the hydrogel again, the hydrogel did not fracture.

In summary, by changing the host and guest monomers and polymers, different synthetic methods can be utilized to design and prepare host–guest complexes according to the different applications. Self-healing hydrogels containing reversible host–guest interactions exhibit some advantages, such as a repeatable healing process without any external energy, long storage time and high healing rate. Self-healing based on host–guest interactions is still a wide field for research due to the diversity of guest molecules and their reversible nature.

#### 2.1.4. Metal Coordination

Metal coordination is a supramolecular structure that introduces metal ions and organic ligands into the matrix. Metal coordination interactions have a wide selection of metal ions (Fe^3+^, Zn^2+^ and Cu^2+^) and ligands (-COOH, -NH_2_ and -OH), which can respond quickly to external stimulation. Meanwhile, their coordination strength and applicability cover a large range of natural and synthetic polymers, thus metal coordination interactions are widely used in the synthesis of self-healing materials. 

Lee et al. [115] modified CNTs with mussel adhesion protein to improve their compatibility with polymer materials and enabled CNTs to be uniformly dispersed in the solution. Then, Fe^3+^ was added to the solution to form a reversible metal coordination with the carboxyl group, which acted as the physical cross-linking point of the system. However, when the hydrogel is damaged, the metal coordination interaction between the carboxyl group and the Fe^3+^ ion at the affected area can be re-established to form a new physical cross-linking point to achieve fast self-healing. In fact, the healing time directly affects the performance of the sensor. Therefore, many researchers have focused on how to shorten the healing time of hydrogels by some methods, such as a self-healing conductive hydrogel that introduces Zn^2+^ and 2,2′:6′,2″-terpyridine (tpy) ligand into a polypyrrole (PPy) matrix with good conductivity by sol–hydrogel conversion [92]. Its excellent conductivity could reach 12 S m^−^^1^. The coordination of Zn^2+^ could connect the separated PPy chains to reform the supramolecular structure and achieve the self-healing of the material after the hydrogel was broken. Self-healing could completely restore its original conductivity at room temperature in 1 min.

In addition to using the method of chemical cross-linking to prepare this type of hydrogel, the approach of physical cross-linking cannot be ignored. Hussain et al. [116] added Fe^3+^ as the cross-linking agent to the physical cross-linking network formed by hydroxyethyl cellulose and PAA. The metal–ligand effectively dispersed energy and improved the mechanical properties of the self-healing hydrogel. In enhancing the mechanical properties of hydrogels, double metal coordination bonds are also used to prepare hydrogels. Shao et al. [117] proposed a physically cross-linked CNF composite hydrogel by a one-pot strategy. Self-healing was achieved by double metal coordination bonds (iron ions and 2,2,6,6-tetramethylpiperidine-1-oxyl (TEMPO)-oxidized CNFs and carboxylate ions on PAA) and hydrogen bonds (PAA and CNF molecular chains). Fe^3+^ and CNFs were used as the cross-linking agents to improve the mechanical properties, such as excellent fracture strength (1.37 MPa), fracture elongation (1803%) and fast self-healing (95.7% recovery ratio within 1 h). Tannic acid (TA) was coated on the surface of nanocrystals (CNCs) by static electricity, and AA polymerization was carried out in situ by free radicals in TA@CNC solution [118]. Aluminum ions were then introduced to form a variety of coordination bonds, as shown in Figure 2d. A nanocellulose-reinforced hydrogel material with a dynamic cross-linking structure and excellent self-healing properties was prepared. The hydrogel could directly adhere to human skin and be used as a wearable electronic sensor to detect large deformations (wrist swings) and weak physiological signals (pulse beats).

### 2.2. Dynamic Covalent Bonds

Repeated self-healing of hydrogels is also possible by forming reversible dynamic covalent bonds in the hydrogel network. Because the bonding strength of dynamic covalent bonds is higher than that of noncovalent bonds, these hydrogels possess good mechanical strength. In addition, these hydrogels also have some other excellent properties, such as pH sensitivity, redox sensitivity and temperature sensitivity. Currently, some dynamic covalent bonds have been successfully utilized to prepare self-healing hydrogels, containing Schiff base linkages, disulfide bonds, boronic/boronate ester bonds and Diels–Alder (DA) reactions. Such covalent links are formed by reversible couplings, and hydrogels are formed via the association equilibrium between rupture and reformation.

#### 2.2.1. Schiff Base Linkage

Schiff base linkage [93,94] is derived from the condensation of carbonyl groups with amines and used as one of the driving forces for self-healing hydrogels. The Schiff base reaction (imine, acylhydrazone bonds) is mediated by the nucleophilic attack of the N atom of the amino group on the electrophilic carbon atom of the aldehyde/ketone, which takes place in an aqueous solution under physiological conditions and generates nontoxic products, ensuring good biocompatibility for Schiff base reaction-based hydrogels. In addition, it has a high chemical reaction selectivity and rapid reaction speed. Once the Schiff base linkages in the network structure are disrupted, the amino or hydrazide groups on the fracture surface rapidly react with the aldehyde groups in contact and form imine or acylhydrazone bonds again, thus reconfiguring the hydrogel matrix for self-repair. It is worth noting that the Schiff base is only stable in an alkaline or neutral environment. 

In recent years, polysaccharides (such as chitosan, hyaluronic acid, sodium alginate, cellulose and dextran) have become ideal matrix materials to prepare self-healing hydrogels with acylhydrazone bonds. This is mainly because their backbones carry a large number of functional groups that can participate in the Schiff base reaction either in a direct way (such as the primary amine groups of chitosan) or after being modified into aldehyde and amine groups. Among them, chitosan is a nontoxic, biodegradable and biocompatible polysaccharide that can only be soluble in acidic aqueous solutions. Therefore, researchers have enhanced chitosan’s water solubility by chemical modifications or conjugations with a specific ligand, making it suitable for the conditions of Schiff base reactions. 

**Figure 3:**
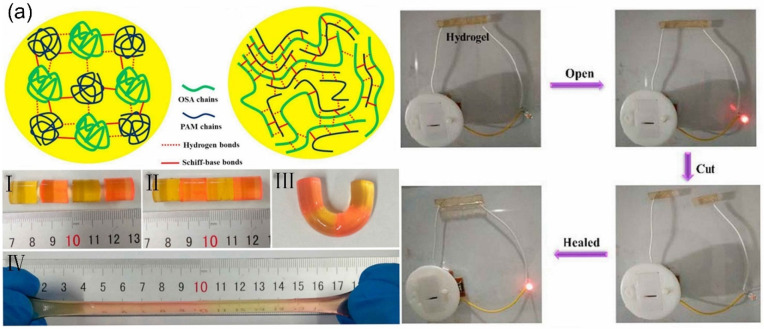

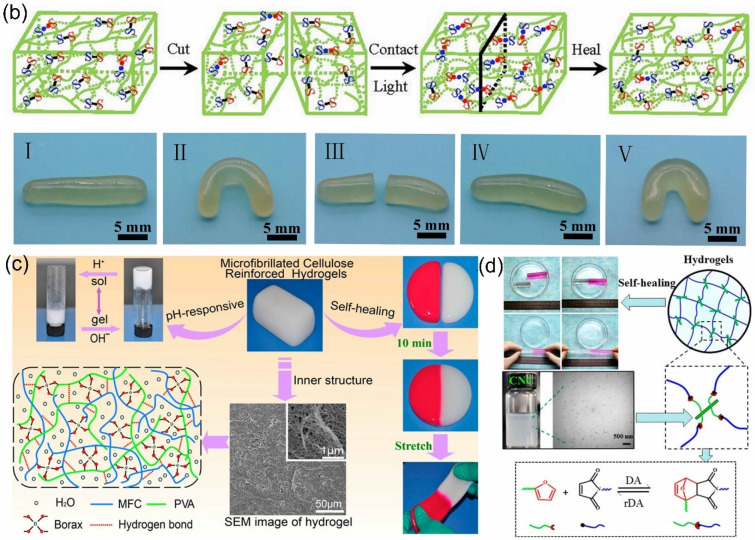
Schematic illustration of self-healing hydrogel and self-healing performance with (**a**) the Schiff base linkage, (**b**) the disulfide bond, (**c**) borate ester bond and (**d**) Diels–Alder (DA) reaction.

Zhang et al. [119] used a large amount of -NH_2_ on chitosan (CS) to condense with benzaldehyde groups on dibenzaldehyde-terminated telechelic PEG to form imine bonds. The study found that this could quickly form a hydrogel within 60 s after contacting CS with telechelic PEG at 20 °C. The self-healing experiment showed that the incision on the hydrogel gradually decreased over time and could be completely healed within 15 min, after the hydrogel broke. The broken hydrogels could self-heal by the dynamic properties of Schiff base linkage. To obtain the self-healing hydrogels with high performances, a self-healing hydrogel formed of oxidized sodium alginate (OSA) and acrylamide (AM) monomer by schiff base reaction was prepared [94]. Figure 3a shows the process of the self-healing hydrogel. The different colored hydrogels were cut into two semicircular hydrogels. Then, the separated semicircular hydrogel was contacted for a period of time, and the fractured surfaces joined together and healed. After self-healing, the hydrogel still retained excellent mechanical and conductivity properties. Yang et al. [120] used modified carboxyethyl cellulose with dibenzaldehyde-terminated PEG under the catalysis of 4-amino-DL-phenylalanine to form a self-healing hydrogel. The prepared hydrogel not only had better self-healing ability but also had dual responsiveness to pH and redox agents. This is because the acylhydrazone bond is more sensitive to pH and the disulfide bond is more sensitive to redox agents. As mentioned in previous studies, the imine and acylhydrazone bonds in Schiff base reaction-founded self-healing hydrogels can be formed under mild conditions and not only allow facile preparation of hydrogels without any stimulation, but also bestow the self-healing ability. Therefore, Schiff base reaction-founded hydrogels prepared through some modification strategies and methods will promote the development and effective application of hydrogels.

#### 2.2.2. Disulfide Bond

The disulfide bond [121] is a dynamic covalent bond based on the thiol/disulfide dynamic exchange reaction, which is sensitive to many factors such as acid, alkali and ultraviolet light. Li et al. [122] proposed a photosensitive cellulose-based self-healing hydrogel by embedding thiuram disulfide bonds into hydrogels via the polyaddition method. The hydrogel could realize rapid self-healing within 2 min, and the cracks disappeared completely under visible light irradiation (Figure 3b). The reason is that the dithiocarbamate ester bonds in the CNC-containing hydrogel could be the result of homolytic cleavage under visible light and produce dormant dithiocarbamyl radical intermediates. When the fracture surfaces were in contact with each other, the dithiocarbamyl radicals broke and recombined on the re-contacted surfaces by exchange and transfer reactions, and the covalent S–S bond was reconstructed to realize the healing of the fracture surface. The self-healing efficiency reached 97%, and the hydrogel could be stretched 42.6 times of the original length. Usually, disulfide bonds are combined with other covalent or noncovalent bonds to enhance the mechanical properties and self-healing efficiency of hydrogels. Dang et al. [95] prepared a healable ionic hydrogel with acrylic acid (AA), choline chloride and ferric chloride through a simple, fast process. The self-healing properties are achieved due to the contribution from disulfide bonds, hydrogen bonds and coordination bonds in the hydrogel. Hydrogels can be used directly as wearable sensors to monitor human movement [123].

#### 2.2.3. Boronic/Boronate Ester Bond

Boronic/boronate ester bonds are formed via a combination of boronic acid and 1,2- or 1,3-diols. These bonds can be formed or broken reversibly depending on the pH or aqueous media. Boronic acid can selectively bond to diols to form boronic esters or boronate esters; therefore, boronic acid can be applied in sensors as a component in drug delivery systems and self-healing materials [96]. 

In polymer networks containing boronic ester bonds, these bonds undergo facile bond exchange via associative or dissociative mechanisms. Lu et al. [124] designed a self-healing hydrogel with boronic ester bonds as the driving force. The process consisted of 3-acrylamidophenylboronic acid (AAPBA) and acrylamide (AM) chain copolymerization and covalently cross-linked with hydroxypropyl guar gum (HPG). The tensile strength increased as the AAPBA, HPG and AM increased. In the hydrogel, the phenylboronic acid groups in AAPBA combined with the 1,3-cishydroxyl moieties of HPG formed dynamic covalent phenylboronic (PBA)-diol ester bonds and endowed the hydrogel with good self-healing properties and tensile properties. The hydrogel (cut into two pieces) could be completely restored for 30 min at room temperature. It was found that the formation of PBA ester bonds was dependent on the pH value. In an acidic environment, AAPBA did not react with HPG, and the hydrogel had poor tensile stress. When the pH was higher than 8.2, stable boronic ester bonds were formed in the hydrogel.

In addition, boronate ester bonds are another dynamic covalent bond formed by free boronic acid and diol. In many studies, borax has been used as an alternative to boric acid, combining with diols to form dynamic B-O bonds, and dynamic B-O bonds are usually regarded as boronate esters. Because the borax can be hydrolyzed in water to form boric acid and borate ions, it has been widely used as a cross-linking agent in the preparation of PVA–borax hydrogels. Lu et al. [125] used the reversible dynamic boronate bond to mix with the microfibrillated cellulose (MFC) obtained by ball milling with borax, and then added a PVA solution to prepare a pH-responsive self-healing hydrogel. The hydrogel containing 3.0% MFC could be stretched 3000%, while the hydrogel without MFC was easily broken. This indicated that MFCs could improve the mechanical properties of the hydrogel. Then, the self-healing process of the two hydrogels was observed, as shown in Figure 3c. After 10 min, the broken hydrogel could be healed. Moreover, the hydrogel was sensitive to pH as it showed a repetitive sol–gel phase transition depending on the pH. 

However, traditional self-healing hydrogels have long self-healing times. Modifying the components of the hydrogels could greatly shorten the self-healing time and increase the conductivity of the hydrogels [96]. In these hydrogels, dynamic boronate bonds were formed by PVA and benzoboric acid groups. The separated hydrogel was contacted for 15 s, and the fractured surfaces could join together and heal together.

#### 2.2.4. Diels–Alder Reaction

The Diels–Alder (DA) reaction [126], also known as diene addition, is the reaction of conjugated diene and dienophile to generate substituted cyclohexene. The DA reaction, as one of the “click chemistry” reactions, plays an important role in the preparation of various functional hydrogels due to its high efficiency, high selectivity and lack of side reactions and byproducts [127]. Additionally, the DA reaction possesses atomic economy and generally requires no catalyst or initiator. Interestingly, the DA reaction is reversible under certain conditions (e.g., at elevated temperature or in organic solvents). Hence, the DA reaction has been used for the preparation of hydrogels. DA reaction-founded hydrogels can be healed by the reversible formation and breakage of covalent bonds upon heating. Specifically, in the damaged hydrogel, the Diels–Alder bonds break upon the application of heat, and the chains become elastic at high temperatures. The elastic chains move to the fracture site to reform the Diels–Alder bonds upon a decrease in temperature, and self-healing occurs as the network is reformed. Shao et al. [97] reported a tough, highly elastic and fast self-healing hydrogel with an interpenetrating network by the Diels–Alder click reaction. The synthesis process of self-healing hydrogels is shown in Figure 3d. The furan group at the crystal end of the modified CNFs and the maleimide at the end of the polyethylene glycol form a thermally reversible covalent bond. As a reinforcing agent and chemical cross-linking agent, CNFs can improve the mechanical properties of the hydrogels.

## 3. Conductive Categories of Self-Healing Hydrogel for Flexible Sensors

Wearable flexible devices are one of the main application fields of flexible sensors, which require the matrix materials of sensors to have good biocompatibility. Currently, most sensor devices are based on inorganic materials (such as metals and silicon) with good conductivity. However, the physical and chemical properties of these inorganic materials are significantly different from those of biological tissues. Inorganic material sensor devices may cause inflammatory reactions in the body when in direct contact with the skin, and the signals collected may be inaccurate. Conductive hydrogels with self-healing properties have shown great potential in sensor devices due to their appropriate electrical and mechanical properties, long service life and good biocompatibility. However, most of the polymer networks in hydrogels are insulated [128,129], so the methods to synthesize conductive hydrogels are as follows: (1) embedding conductive fillers into an existing nonconductive hydrogel matrix; (2) constructing hydrogel networks by self-polymerization or self-assembly of conductive polymers; and (3) diffusing free ions.

Conductive hydrogels with self-healing properties can significantly prolong the service time of electronic devices. Many conductive hydrogels with high self-healing properties are based on the intrinsic repair method by designing reversible (weak) interactions in polymer networks. Under low external stress, the weak bonds can break first and adsorb energy to protect the covalent polymer network. When the covalent polymer network of the hydrogel is damaged under a higher external stress, the reversible bonds will reform to restore the properties of the hydrogel.

### 3.1. Self-Healing Hydrogel with Conductive Fillers

The most convenient way to obtain a highly conductive self-healing hydrogel is to embed conductive fillers into an existing nonconductive hydrogel matrix. These conductive fillers are suspended in the hydrogel precursor solution for polymerization and cross-linking to form a conductive network in the hydrogel. Typically, conductive fillers include metal nanomaterials, carbon nanomaterials, transition metal carbides and carbonitrides. As the nanoscale conductive filler can be uniformly dispersed into the hydrogel matrix, stress concentration is avoided, enabling the mechanical strength of the hydrogel to be significantly increased. In addition, the type and content of conductive fillers used in synthetic self-healing conductive hydrogels, as well as the surface modification and cross-linking methods used, have a significant impact on the properties of hydrogel sensors, such as conductivity, stretchability, toughness, fatigue resistance and self-healing properties [130,131]. However, the conductive mechanism of conductive filler-based hydrogels is complex, and different conductive mechanisms need to be combined to explain this conductive phenomenon. Currently, the conductive mechanisms commonly used for conductive filler-based hydrogels are contact conduction and tunnel conduction [132]. As the filling amount of conductive filler increases, the conductivity gradually increases. When the filler content reaches the critical volume fraction, the conductivity increases sharply. This phenomenon is called “percolation threshold”. However, as the filler content continues to increase, the conductivity no longer increased significantly [133]. In conductive filler-based hydrogels, in one case, conductive fillers are in contact with each other to form conductive network pathways, and in the other case, conductive fillers are not in contact with each other, and they exist in isolation or in aggregates. However, when the spacing between the conductive fillers is very small, the electrons of the conductive filler may be activated by a thermal vibration due to the interaction between the conductive filler particles. The activated electrons absorb energy and jump across the barrier of the thin polymer layer to the adjacent conductive filler, thereby forming a tunneling current and conducting path. This is the electron tunneling effect conductive mechanism [134].

#### 3.1.1. Metal-Based Nanomaterials

Metal nanomaterials (such as metal and its oxide nanoparticles, nanowires and nanorods), as one type of the preferred raw materials for the preparation of functional conductive hydrogels, have high conductivity, optical properties, catalytic properties and easy processing [135,136,137,138,139]. Contact conduction theory can explain the conductive mechanism of metal-based hydrogels. When the content of metal filler is below the permeation threshold, only a local conductive network can be formed inside the hydrogel and its conductivity is low. As the filler content gradually rises above the percolation threshold, a complete conductive network is formed inside the hydrogel and the conductivity increases significantly.

**Figure 4:**
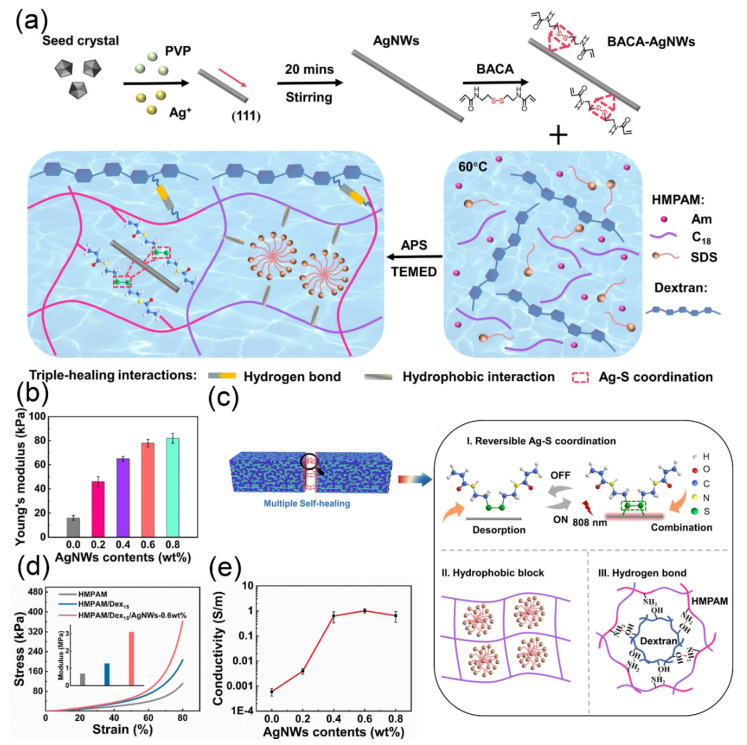
Metal-based nanocomposite hydrogels. (**a**) Schematic for preparation of nanocomposite hydrogels. (**b**) Young’s modulus of the nanocomposite hydrogels with different AgNWs contents. (**c**) Self-healing mechanism of prepared hydrogels in this work. (**d**) Compression curves of the different hydrogels, respectively. (**e**) Conductivity of the nanocomposite hydrogels with different AgNWs contents.

Incorporating metal nanoparticles into hydrogels improve their strength but causes nonbonding between fillers and the polymer matrix. To address this issue, some researchers have proposed solutions. He et al. [140] fabricated a conductive hydrogel made of PVA and in situ reduced Au nanoparticles, which could achieve self-healing without external stimuli due to hydrogen bonding and reversible metal–ligand coordination. Furthermore, the conductive hydrogel has a high mechanical toughness (maximum compressive strength was 7.26 MPa). To obtain a shorter healing time and better conductivity, the silver/reduced graphene oxide (Ag/rGO) composite material was combined into PVA–borax [141]. This hydrogel contains many hydrogen bonds and can heal itself within 3 s without any external stimulation at room temperature. Ding et al. [142] proposed a self-healing hydrogel-based sensor with conductivity, antibacterial and self-healing properties consisting of hydrophobic modified polyacrylamide (HMPAM), bis (acryloyl) cystamine (BACA)-modified silver nanowires (AgNWs) and dextran, as shown in Figure 4a. This sensor had ultralow strain (0.05%), a wide strain sensing window (0.05–1200%), a wide operating frequency range and superior cycle stability (200 relatively low resistance changes). The Young’s modulus of the hydrogels increased with the increasing ANGWS content, as shown in Figure 4b. However, these hydrogels with different contents of AgNWs exhibited quite a low Young’s modulus (10~90 kPa) and were very sensitive to strain signals. Meanwhile, Figure 4c shows a number of reversible noncovalent and hydrogen bonds contained in the hydrogel network, as well as reversible Ag–S coordination bonds, which were helpful to improve the self-healing and mechanical properties of the hydrogel. Furthermore, Figure 4d shows that HMPAM/Dex/AgNW nanocomposite hydrogels have outstanding compression performance. According to the conductivity experiment, the conductivity increased with AgNW content and reached a maximum of 1.0 S m^−^^1^ (Figure 4e). In particular, the sensor was first realized to recover its sensing properties after self-healing.

Although AgNWs have excellent conductivity and easy processing, research on hydrogels filled with AgNWs is still very limited, mainly because (i) the mechanical properties of the hydrogel are reduced when the filled AgNWs are not uniformly dispersed in the hydrogel polymer matrix, (ii) the process of patterning AgNWs on the hydrogel surface is less and (iii) there is weak interfacial bonding between AgNWs and the hydrogel matrix. Zhu et al. [143] proposed an easily patterned, highly conductive self-healing hydrogel sensor by dispersing AgNWs in a highly viscoelastic hydrogel matrix. The mechanical properties of this hydrogel were superior to those of other hydrogels, and the fracture stress could reach 3.3 MPa. The hydrogel sensor had a gauge factor of 58.2 and could detect human motions.

Therefore, the introduction of metal nanomaterials into hydrogels can effectively improve the conductivity and mechanical properties [144,145]. However, precious metal conductive materials (such as gold and platinum) are usually expensive, which severely limits their large-scale utilization. In addition, metals are prone to corrosion in a humid environment, resulting in a decline in the electrical properties of hydrogels, which greatly hinders their potential application in the field of bioelectronics.

#### 3.1.2. MXene-Based Nanomaterials

MXene (transition metal carbides and carbonitrides) nanosheets [146], as a new two-dimensional material, have some excellent properties: high conductivity, good mechanical properties and water solubility. It is widely used in the fields of supercapacitors [147,148] and sensors [31]. Because MXene has abundant hydrophilic groups, these hydrophilic groups firmly combine MXene nanosheets with the hydrogel network through multi-physical interactions, thereby improving the mechanical properties [149,150]. In addition, MXene nanosheets have good water solubility, which allows them to be evenly distributed in the hydrogel network, not easily agglomerate and form a stable conduction network [151].

**Figure 5:**
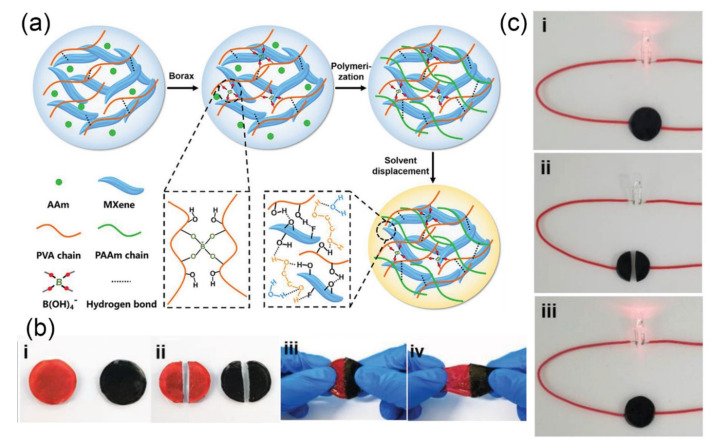
MXene based nanocomposite hydrogels. (**a**) Schematic illustration of the fabrication of the self-healing hydrogel. (**b**) The self-healing behavior between two pieces of hydrogel: (**i**) original, (**ii**) completely bifurcated, (**iii**) self-healed, and (**iv**) stretched after healing. (**c**) A circuit comprising (conductive MXene nanocomposite organohydrogel) MNOH in series with a red LED indicator: (**i**) original, (**ii**) completely bifurcated, (**iii**) self-healed.

According to the contact and tunnel conduction theory, the conductivity of the MXene-based hydrogel increases with increasing the MXene nanosheets content, reaching an optimum conductivity after exceeding to the percolation threshold. Combined with effective polymer action, an effective tunneling current is achieved for the MXene nanosheets, resulting in a high conductivity of the hydrogel. Simultaneously, the deformation of the hydrogel causes a change in conductivity. Specifically, under tensile deformation, the spacing between the MXene nanosheets in the hydrogel increases, which induces a decrease in conductivity. In contrast, under compressive deformation, the distance between the MXene nanosheets decreases, which increases the conductivity of hydrogels. As such, Zhang et al. [152] added MXene nanosheets (Ti3C2Tx) to a matrix containing PVA, water and anti-dehydration additives, and the obtained hydrogel had excellent tensile strain sensitivity, self-healing properties and conductivity. The self-healing properties of the hydrogel were achieved by hydrogen bonding. It was found that the value of the compression experiment was higher than the values of the tensile experiment. Some movement directions and speeds of the sensor surface could be detected more accurately by this asymmetric sensitivity. A self-healing hydrogel suitable for low temperature was also proposed [153]. The hydrogel polymer network was composed of PVA, MXene nanosheets and polypropylene amine (PAAM) (Figure 5a). MXene nanosheets were added to the hydrogel matrix to form a three-dimensional conductive network, which contributed to electron transmission and made the hydrogel have excellent conductivity. The self-healing process of the hydrogel is shown in Figure 5b. The two semicircular hydrogels with different colors healed together by dynamic cross-linking and molecular interactions. The healed hydrogel did not break when stretched again. Meanwhile, to further investigate the effect of self-healing on the electrical performance, a circuit with a red LED indicator was designed (Figure 5c). When the hydrogel was cut off completely in the circuit, the red LED indicator switched off immediately. After the two fractured parts were re-contacted and healed, the circuit was restored and the red LED indicator lit up again. A wider strain range contributes to expanding the applications of hydrogels. Wei et al. [154] proposed a ternary hybrid network hydrogel composed of TA-modified CNFs, which combined the conductive MXene nanosheet network and covalently cross-linked PAAM network. It contained a large number of hydrogen bonds and dynamic borate bonds, which not only realized the self-healing properties of the hydrogel, but also improved the tensile properties of the hydrogel. The hydrogel also had a wide working strain range and high sensitivity, which was suitable for human body motion monitoring.

However, there are still many problems in the application of MXene-based flexible hydrogels. For example, some dangerous chemicals are used in the preparation of MXene nanosheets, which may pollute the environment and introduce harmful substances. The prepared MXene-based flexible hydrogel has a relatively weak network and excellent mechanical properties, which will limit its application [155,156]. Therefore, to make MXene nanosheets more widely used in hydrogel sensors, it is necessary to perfect the preparation method of MXene nanosheets.

#### 3.1.3. Carbon-Based Nanomaterials

Carbon nanotubes (CNTs) [157,158,159,160], graphene oxide (GO) [161,162], carbon fiber [163] and other carbon-based nanomaterials [164,165,166] are the preferred conductive materials with great development prospects due to their unique properties (such as high conductivity, relatively high Young’s modulus, environmental stability and good biocompatibility). The excellent stability of carbon-based nanomaterials in humid environments greatly promotes their application in conductive nanocomposite hydrogels (Table 2), making them a good substitute for metal nanomaterials [167]. 

**Figure 6:**
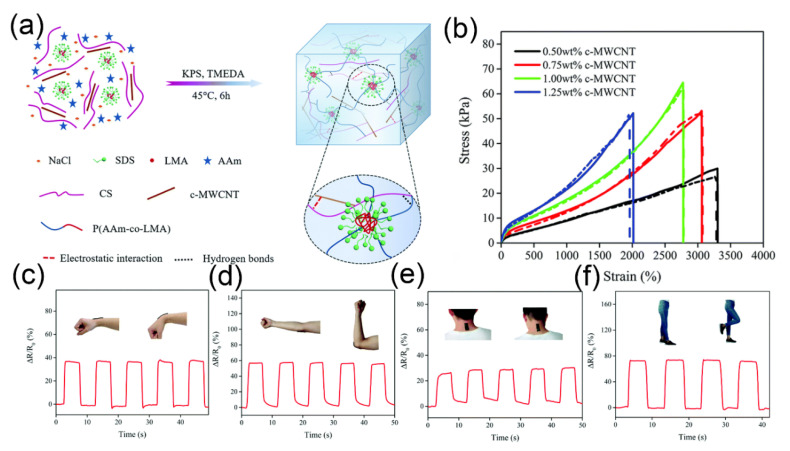
CNTs-based nanocomposite hydrogels. (**a**) Formation of the HPAAm/CS-c-MWCNT hybrid hydrogel. (**b**) The effects of c-MWCNT content on the self-healing behavior of the hybrid hydrogels. The relative resistance variations of the hydrogel in response to human motions: (**c**) wrist, (**d**) elbow, (**e**) neck and (**f**) knee joint bending and releasing, respectively.

The aspect ratio of CNTs exceeds 1000, which can allow them to achieve electron transfer at lower voltages [168]. With increasing the CNT content in the hydrogel system, the conductivity of the hydrogel also increases [169]. Here, contact conduction and tunneling conduction can be used to clarify the conductive mechanism of hydrogels. However, the random distribution and easy aggregation behavior of CNTs limit the properties of hydrogels. The modification of CNTs is the key to overcoming the poor compatibility between CNTs and the polymer matrix [170,171]. Han et al. [172] reported a multi-functional conductive hydrogel based on PVA–borax and CNT–CNF composite materials, in which borax was used as a cross-linker to make the hydrogel mechanically tough and self-heal. When the CNTs content was below 0.3 wt%, no complete conductive network was formed within the hydrogel, which resulted in low conductivity of the hydrogel. When the CNTs content was increased to 0.5 wt%, the conductivity of the composite hydrogel increased rapidly to 8.0 ± 0.5 S m^−1^. The results show that the permeation threshold of CNT content was 0.3 wt%. As the content of CNTs continued to increase, conductive channels were formed within the hydrogel, thus enabling the contact conduction mechanism. In addition, cellulose nanofibers (CNFs) cannot only act as a dispersant to stabilize the dispersion of CNTs in hydrogels, but also achieve an effective tunneling current for CNTs to achieve high conductivity at low CNTs content. In this hydrogel network, borax formed borate ester bonds with PVA chains, and CNFs contributed to its rapid self-healing ability. Similarly, it is still feasible to use these two conductive theories to explain the conductive phenomena of carbon-based hydrogels. Wang et al. reported a conductive self-healing hydrogel with adhesion properties by adding dopamine (DA) [173]. Furthermore, it was found that MWCNTs could be uniformly dispersed in the hydrogel network due to π–π interactions between DA and MWCNTs. In addition, multiple hydrogen bonds were formed to realize the rapid self-healing of the hydrogel. The hydrogel also exhibited good adhesion in the presence of DA, which improved the comfort of the sensor. In addition, Gao et al. [174] proposed a multifunctional conductive hydrogel composed of a PAM/CS composite network, which is shown in Figure 6a. The PAM network was cross-linked by hydrophobic associations, and the CS network was ionically cross-linked by MWCNTs. These two networks were further interconnected by physical entanglement and hydrogen bond interactions. Because the dynamic cross-linking network effectively dissipated energy, the prepared hydrogel exhibited excellent flexibility, adhesion and self-healing. After re-contacting the two cut samples of hydrogel for 48 h, they were completely self-healed. Moreover, for the hydrogels with different c-MWCNT contents after healing for 48 h, the tensile curves coincided with those of the original samples, as shown in Figure 6b. (The solid lines represent the tensile curves of pristine hydrogel, and the dashed lines are the tensile curves of hydrogel after healing for 48 h.) The conductivity of this hydrogel increased dramatically as the c-MWCNT increased from 0.5 wt% to 1 wt%. When the content of c-MWCNT was increased to 1.5 wt%, the increase in the conductivity of the hydrogel was not significant. The disadvantage is that the tensile properties of the hydrogel deteriorate, although increasing the content of c-MWCNT increases the conductivity of the hydrogel. Significantly, the hydrogel was simply assembled as a wearable sensor. It can accurately monitor human motions, such as elbow, neck and knee joint motions, as shown in Figure 6c–f. 

Graphene oxide (GO) nanosheets contain abundant hydroxyl, epoxy and carboxyl groups on their surface and have been used as cross-linkers to prepare conductive hydrogels. A copolymer hydrogel double-cross-linked by laponite and GO could achieve repeated healing [175]. The hydrogel as an electrolyte in supercapacitor not only had ultrahigh mechanical tensile properties of 1000% but could also achieve repeated healing under infrared light irradiation and heating conditions. Xia et al. [176] prepared a conductive self-healing hydrogel with a physical cross-linking network. Using the FeCl_3_ as a cross-linking site, the hydrogel was formed with PAA, CS and GO in a solvent mixture of water and glycerol. The conductivity of PAA/CS/GO/Gly hydrogel could reach 5.6 ± 0.25 × 10^−3^ S cm^−1^, which is attributed to the effect of GO and ions (Fe^3+^, Cl). These hydrogel sensors also had a rapid response time (40 ms) and moderate gauge factor (GF) (1.138). In addition, the hydrogel could be self-healed rapidly due to coordination interaction and hydrogen bonds. After 1 h of being self-healed, the stretch curve of the healed hydrogel was almost identical to the original sample. Wang et al. [177] prepared a new self-healing conductive hydrogel with a fast self-healing ability and good conductivity (10.5 mS dm^−1^). This hydrogel was synthesized by GO, soluble starch and poly (sodium 4-vinyl-benzenesulfonateco-N-(2-(methacryloyloxy)ethyl)-N,N-dimethylbutan-1-aminium bromide)(P(NaSS-co-MOBAB)). It is worth noting that the hydrogel conductivity could be restored after self-healed. The experimental tests have shown that after 10 cut-healing cycles, the hydrogel could be restored to 80% of its original conductivity.

**Table 2:** Summary of properties of recently reported carbon-based hydrogel sensors.

Hydrogel Materials	Conductive Type	Self-Healing Mechanism	Gauge Factor	Conductivity	Application	Ref
PC/rGO/PVA	Electron	cross-linked bonds	14.14		Wearable E-skin	[178]
PVA/PDA/pRGO	Electron	hydrogen bonds		2.7 S cm^−1^	soft strain sensor	[179]
PVA/CNTs/ graphene	Electron/ion	hydrogen bonds, borate ester bond	52.7		electronic device	[180]
TOCNF/GN/PAA	Electron/ion	hydrogen bonds, metal-ligand interactions	5.8	2.5 S m^−1^	soft sensor devices	[181]
P(DMA-co-PFPA)/ SWCNTs/PVA	Electron/ion	dynamic boronate ester bonds		1.27 S m^−1^	electronic skins	[182]
rGO–SAP	Electron/ion	hydrogen bonds		1500 ΩM	soft sensor devices	[183]
PVA/PDAP/ MWCNT	Electron	borate bonds, hydrogen bonds			wearable electronics	[184]
PAA/CS/GO/Gly	Electron/ion	hydrogen bonds, electrostatic interaction	1.138	5.6 × 10^−3^ S cm^−1^	wearable sensor	[176]
PAA-GO	Electron/ion	coordination crosslinking, hydrogen bonds	0.46		wearable sensor	[162]
EW/CNT	Electron/ion	hydrogen bonds, hydrophobic interactions		87.8 kΩ	epidermal sensors	[185]
CS/ZnPcTa	Electron	Schiff-base linkage		0.0029 S cm^−1^	biomedical applications	[186]
PVA/Gly/CB/CNT	Electron	hydrogen bonds	2.1		wearable sensor	[187]
Poly(NIPAM-co-β-CD)/CNT/PPY	Electron	Host−Guest Interactions		34.93 S m^−1^.	wearable sensors	[188]
CS/DA/GO	Electron	hydrogen bonds, π-π stacking		1.2 × 10^−3^ S cm^−1^	engineering applications	[189]
rGO/AM	Electron	covalent bonds hydrogen bonds		27.2 S m^−1^	artificial skin, soft robotics	[190]
PNIPAM/Laponite/CNT	Electron	electrostatic interaction, hydrogen bonds		0.17 S m^−1^	wearable sensor	[191]
PAM/MWCNTs	Electron	hydrophobic interactions, hydrogen bonds	5.6	0.5 S m^−1^	Wearable medical monitoring	[192]
GOxSPNB	Electron/ion	electrostatic interaction, hydrogen bonds		10.5 mS dm^−1^	conductive adhesive materials	[177]
PAA/GO/Ca^2+^	Electron/ion	Hydrogel bonds, ionic interactions		257.31 kΩ	wearable biosensors	[193]
AlgPBA/PVA/ PAM/rGO	Electron	covalent ester bonds, hydrogen bonds		0.0525 S m^−1^	E- skins, healthcare monitoring,	[194]
PVA/FSWCNT/ PDA	Electron/ion	Hydrogen bonds, π–π stacking,			wearable sensors.	[195]

Abbreviation: proanthocyanins (PC), acrylamide (AM), Partially reduced graphene oxide (pRGO), functionalized single-wall carbon nanotube (FSWCNT), carbon black (CB); 2,2,6,6-tetramethylpiperidine-1-oxyl oxidized CNFs (TOCNFs), graphene nanocomposites (GN), Egg white (EW), Sodium polyacrylate polymer particles (SAP), β-cyclodextrin (β-CD); zinc phthalocyanine tetra-aldehyde (ZnPcTa), glycerol (Gly), Pentafluorophenyl acrylate (PFPA) Nisopropylacrylamide(NIPAM), N,N-Dimethyl acrylamide (DMA).

### 3.2. Self-Healing Hydrogel with Conductive Polymers

Hydrogels prepared by incorporating a conducting polymer into a hydrogel matrix typically have excellent conductivity. The conductive polymers (CPs) include PANI [196,197,198], PPy [199] and poly (3,4-ethylenedioxythiophene): polystyrene sulfonate(PEDOT:PSS) [200,201,202,203,204]. However, the conjugated structures of conductive polymers are inherently rigid and natively hydrophobic, which make them incompatible with the hydrophilic polymer matrix, resulting in the conductive component tending to aggregate and inhomogeneously distribute. The insufficient weak interactions between the two ingredients usually result in the weak mechanical performance of the hydrogel and poor adaptability to large deformations, which seriously impede practical applications in the fields of wearable strain sensors. Additionally, conductive polymers in the intrinsic state, the π electrons on the conjugated structure, are difficult to migrate along the long chain of the molecule when unexcited, so the conductivity is limited and needs to be modified by chemical doping [205]. Specifically, the essence of doping is that the polymer chain with a conjugated structure has a charge transfer or redox reaction with the dopant, so that the formed electrons can move along the direction of the molecular chain, and the conductivity of the polymer will be significantly improved [206]. The conductive mechanism of the conductive polymer is related to the type of dopant, which can generally be divided into a charge transfer mechanism and proton acid mechanism [207,208].

The one conductive mechanism of hydrogel is the charge transfer when conductive polymers are modified with oxidizing dopants, such as metal salts (FeCl_3_) and halogens (I_2_, Br_2_). For example, FeCl_3_ acts as a p-type dopant, taking electrons from the large π bonds of the polymer, which reduces the hindrance of hole-electron migration, thereby increasing the conductivity [209]. Ding et al. [210] reported a new strategy for the design and preparation of a multifunctional hydrogel. Specifically, PPy was assembled onto the surface of CNFs and then mixed with PVA/boric acid solution. The results show that the PPy was well dispersed in the hydrogel to form a continuous conductive network, which promoted the tunneling of charges transferred between adjacent PPy chains. The conductivity of the hydrogels was increased from 1.5 to 4.8 S cm^−1^ with increasing PPy. The obtained hydrogel exhibited a high water content (∼94%), low density (∼1.2 g cm^−3^) and rapid self-healing ability.

Another conductive mechanism is the protonic acid mechanism. Commonly used proton acid dopants are HCl, H_3_PO_4_ and H_2_SO_4_ or other non-oxidized Lewis acids (BF_3_) [211]. Specifically, there is no migration of electrons between the polymer chain and the dopant. However, the proton of the dopant is attached to the carbon atom of the main polymer chain, causing a change in the charge distribution on the polymer chain [212]. Typically, hydrogels are prepared by free radical polymerization [213,214,215]. Polymer monomers, oxidizing agents (APS) and/or dopants are used to complete the polymerization and are homogeneously dispersed into the hydrogel matrix by the cross-linking agents to form a complete conductive network. Yang et al. [216] proposed a self-healing hydrogel with good extensibility, using trypan blue (TB) as the cross-linking agent to form a semi-interpenetrating network with PAA and PPy. In addition, the PAA support structure and the PPy molecular chain can be well connected to form an interconnected conductive network by the large π conjugated ring of TB. So, the conductivity of this hydrogel was equivalent to that of pure PPy hydrogel, up to 15 S m^−1^. Its elongation at break exceeded 750%. In addition, the broken hydrogel could recover more than 60% within 10 s. A self-healing hydrogel can also have antibacterial properties by PPy and Zn-functionalized CS molecules cross-linked with PVA [217]. The conductive component CS-PPy was synthesized by graft polymerization of PPY on double bond-decorated chitosan with a free radical. When the content of CS-PPy was 1%, the maximum conductivity of the hydrogel reaches 1.16 S cm^−1^. The reason for this phenomenon was that the content of the conductive component reached the electrical percolation threshold and the PPy molecular chain formed a connected conductive network in the hydrogel. In hydrogels, multiple covalent bonds (hydrogen bonds and zinc-based coordination bonds) also endowed the hydrogel with self-healing properties. In addition, the introduction of Zn ions enhanced the antibacterial properties of the hydrogel. 

**Figure 7:**
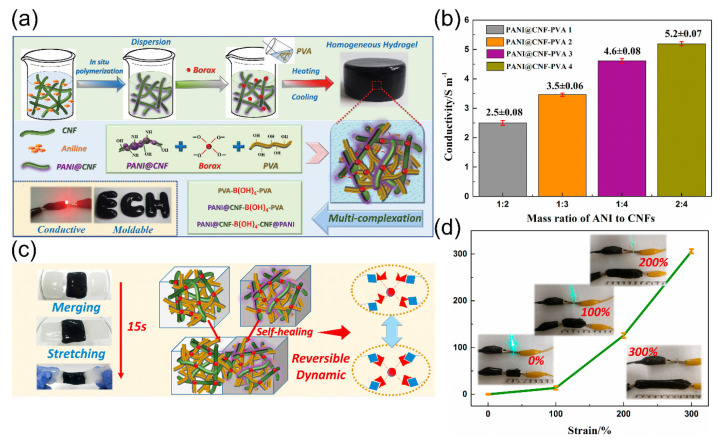
Conductive polymer hydrogel with PANI. (**a**) Schematic for the preparation of PANI/CNF/PVA composite hydrogels. (**b**) Conductivity variation of PANI/CNF/PVA gels with changed mass ratio of ANI monomers to CNFs. (**c**) Illustration of self-healing process of hydrogels. (**d**) Luminance change of a green LED and the relative resistance change of hydrogels under varying strains.

In situ polymerization of conductive polymer monomers onto nanostructured flexible templates (CNF, CNC) to synthesize hydrogels with a stable, flexible, continuous conductive network thereby improves electrochemical and mechanical properties of the hydrogels [218,219,220,221]. Han et al. [222] prepared PANI/CNF nanocomposites by dispersing aniline on CNFs by in situ polymerization. Then, the nanocomposite was introduced into the borax cross-linked PVA hydrogel to prepare a self-healing hydrogel with good ductility and excellent conductivity (Figure 7a). As shown in Figure 7b, with the mass ratio of aniline (ANI) monomers to CNFs increased, the hydrogel conductivity increased from 2.5 to 5.2 S m^−^^1^; the result shows a nonlinear enhancement of conductivity with increasing the PANI content. It was also demonstrated that the PANI/CNF complex was well dispersed in the PVA system, resulting in the construction of an effective conductive pathway. The hydroxyl groups and dynamic reversible cross-linked bonds in the hydrogel network enable the hydrogel to recover quickly within 15 s, as shown in Figure 7c. Figure 7d shows that the electrical pathways inside the hydrogel are still maintained during the stretching process, indicating the high stability and stretchability of the hydrogels. In addition, Song et al. [223] added CNC–PANI polymer (in situ polymerization) into PVA/borax to prepare the hydrogel. The separated hydrogel could recover quickly without any external stimuli under the effect of hydrogen bonds and dynamic borate bonds. The sensor made from this hydrogel could be sensitive to tiny movements of the human body (swallowing, bending of fingers or joints).

### 3.3. Ionic Self-Healing Hydrogel

The hydrogel is composed of a three-dimensional framework and significant water molecules (water content above 90%). Such a structure provides many channels for ion migration, which provides the possibility to synthesize excellent ion conductive hydrogels. The preparation method of ion conductive hydrogels usually incorporates inorganic salts (e.g., LiCl, NaCl or KCl) [224,225] into the hydrogel network, balancing the conductive properties and mechanical properties of the hydrogels [226]. Inorganic salts are a strong electrolyte, which are dissolved in water to form freely moving anions and cations. Under the action of an electric field, the positive ions generated within the hydrogel move in the direction of the electric field and the negative ions in the opposite direction, enabling rapid transport of ions and giving the hydrogel a good ionic conductivity [227].

**Figure 8:**
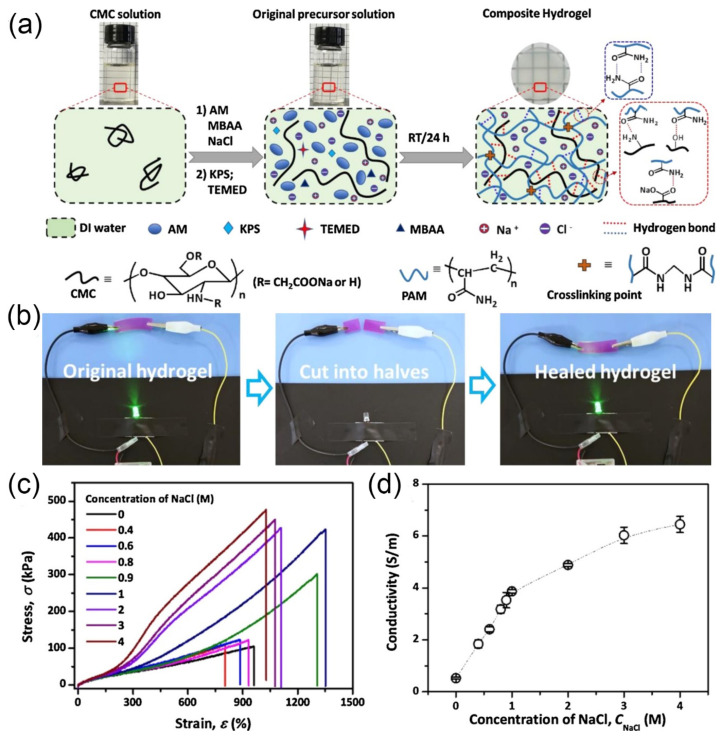
Ionic self-healing hydrogel with inorganic salts. (**a**) Schematic for the preparation of PAM/CMC/NaCl composite hydrogels. (**b**) Conductivity diagrams of hydrogel before and after healing. (**c**) Tensile stress–strain curves of the composite hydrogels prepared with various C_NaCl_. (**d**) The conductivity of the hydrogels prepared with various content of NaCl.

Extensive research has shown that the mechanical strength of polyvinyl alcohol-based or chitosan-based hydrogels can be significantly improved by using the salting out effect of NaCl. Currently, the method used is to soak the prepared hydrogels in NaCl solution. For instance, a self-healing hydrogel with a semi-interpenetrating polymer network using carboxymethyl CMC, NaCl and PAM is shown in Figure 8a [228]. When the concentration of NaCl reached 0.9 M (unsaturated), the salting out effect could occur in hydrogel; thus, it caused more hydrogen bonds to form between the CMC and PAM chains. During the stretching process, the hydrogen bonds act as sacrificial bonds to dissipate energy, resulting in a significant enhancement of the mechanical properties of the hydrogel. Moreover, the NaCl introduced in the hydrogel promoted the formation of hydrogen bonds between the carboxymethyl CS and PAM chains and subsequently improved the mechanical properties (Figure 8b) and self-healing properties of the hydrogel (Figure 8d). In addition, the NaCl solution gives the hydrogel better properties (water retention and freezing resistance). Yuan et al. [229] proposed a PAA/2-hydroxypropyltrimethyl ammonium chloride chitosan (HACC) self-healing hydrogel by in situ polymerization in NaCl solution. The hydrogel showed excellent mechanical properties (the fracture stress was 3.31 MPa, the Young’s modulus was 2.53 MPa and the compressive stress was 60 MPa). Putting the broken hydrogel into NaCl solution, the self-healing efficiency reached 61%. In addition, the hydrogel was rich in sodium and chloride ions and had high ionic conductivity. This ion conductive hydrogel with salts is believed to be an ideal material for the fabrication of strain sensors [230,231,232,233].

LiCl and KCl are also important components for preparing ionic self-healing hydrogels. Lv et al. [234] reported a dopamine-functionalized hyaluronic acid (HAC)/borax/PAM self-healing hydrogel. Abundant conductive ions were formed in the hydrogel network by introducing LiCl solution, which improved the conductivity of the hydrogel. In addition, Wu et al. [235] prepared a KCL/PAM/carrageenan self-healing hydrogel. The ethylene glycol/glycerol binary solvent introduced into the hydrogel formed a strong hydrogen bond with water molecules so that the hydrogel had excellent self-healing properties. The hydrogel also had good freezing resistance and drying resistance due to the existence of this binary solvent [236,237,238].

In summary, introducing conductive particles and conductive polymers into hydrogels can establish their conductive network. To improve the conductivity of hydrogels, more conductive fillers need to be added. However, during the stretching process, the interconnected conductive polymers or overlapping nanomaterials will be irreversibly separated from each other, resulting in a significant decrease in conductivity. Therefore, scientific researchers turned their attention to the direction of preparing ion-conducting hydrogels. Ion-conducting hydrogels not only have better ductility and are suitable for sensors in more situations but can also be used in the field of energy storage, broadening the application range of hydrogels. In addition, ion-conducting hydrogels also have good biocompatibility, which gives them great potential in the field of biomedicine. 

## 4. Conclusions and Perspective

Hydrogel-based flexible sensors have rapidly developed in wearable electronic devices, electronic skins, artificial intelligence and other popular areas due to their high sensitivity and conductivity, strong tensile properties and excellent mechanical properties. To improve the lifetime of the hydrogel-based flexible sensor, it is necessary to introduce self-healing properties that can repair structural damage and restore the sensing ability in the hydrogel to resist fracture damage under continuous action. Moreover, for hydrogel flexible sensors, conductivity is another important property. This review summarizes the latest research status and research progress in self-healing hydrogel-based flexible sensors, including the self-healing mechanism and conductivity. Self-healing performance is the best solution for flexible sensors when dealing with damage and chapping. Many factors should be coordinated while improving the conductivity of self-healing flexible materials, such as achieving ultrahigh conductivity while maintaining the flexibility, elasticity, repairability and high transparency of flexible materials. Although great progress has been made in the preparation and research of self-healing conductive hydrogels, which are widely used in artificial skin, artificial intelligence, flexible sensors, wearable devices and other fields, self-healing conductive hydrogels still have limitations and face many challenges, which as described as follows

(1) High conductivity, mechanical and tensile properties are the basic requirements for most hydrogel-based sensors. However, as wearable sensors, direct attachment to the skin surface is required for practical applications, requiring the development of conductive hydrogels with additional features, such as self-healing and tissue adhesion. The current research is focused on the design and preparation of hydrogels with self-adhesive properties through the addition of polysaccharides, proteins, polyethylene glycols and polydopamine (PDA). Self-adhesive hydrogels prepared by adding polysaccharides, proteins and polyethylene glycols have good biocompatibility, but these hydrogels have poor adhesion and toughness. The strategy has largely enhanced the adhesion of hydrogels by adding PDA, but dopamine is easily oxidized, resulting in a dopamine-based hydrogel whose adhesion is not sustainable and repeatable. In the future, the mussel adhesion mechanism should be investigated in depth, such as the interaction between mussel proteins and the effect of multiscale structure on the adhesion mechanism, to optimize the self-adhesive properties of the hydrogel-based sensor.

(2) Although many reports mention good self-healing properties in their studies, they have not been evaluated fully and accurately. Therefore, there is a need to establish standardized experimental evaluation criteria to measure these properties. For instance, in hydrogel self-healing tests, the volume size of the hydrogel, the number of cuts, the healing time and the characterization after healing all need to be accurately represented.

(3) In addition to the material aspects, we will focus more on the packaging, integration and practical applications of hydrogel-based sensors. While several strategies, such as surface modification and encapsulation, have been proposed to address these issues, these strategies can affect the mechanical properties of the hydrogel and reduce the conductivity and sensitivity of the sensor. Future research on the integration of hydrogels should take into account the interface difference between the “soft” material and the “hard” encapsulation material, the comfort of the encapsulated hydrogel sensor and the fatigue resistance and durability of the encapsulation material.

Thus, the development of a flexible sensor with excellent comprehensive properties, low cost and a simple process will be of great significance to the further development of wearable electronic devices.

## Figures and Tables

**Table 1:** Comparison of the different performances of self-healing hydrogels.

Hydrogel Materials	Self-Healing Systems	Self-Healing Time	Mechanical Healing Efficiency	Mechanical Property Recovery	Ref.
PAAm/SDS/NaCl/C18-C22	noncovalent	a few seconds	~100%	break at elongation ratios	[85]
κ-CG/PAA	noncovalent	24 h	67%	toughness	[86]
HPAAN/PDA	noncovalent	5 h	49%, 67%, 78%	mechanical strength, tensile strain, modulus	[87]
XG/MMT/PAAm	noncovalent	24 h	70%	tensile	[88]
PVA/Agar/AS	noncovalent	--	27.8%, 82.9%	tensile stress, tensile strain	[89]
f-BNNS/clay/PNIPAM	noncovalent	6 h	~70%	tensile	[90]
βCD-Ad	noncovalent	24 h	84%	strength	[91]
PPy/G-Zn-tpy	noncovalent	60 s	~100%	strength	[92]
PVA/AMCS7/ADA	covalent	12 h	--	--	[93]
OSA/PAM	covalent	6 h	>70%	tensile strength	[94]
LA/PAA/Fe^3+^	covalent	14 h	86%	fracture stress	[95]
PVA/SA/NaCl	covalent	15 s	well restored	heavy object pull test	[96]
CNC/PEG	covalent	24 h	78%	tensile strength	[97]

Abbreviation: κ-carrageenan (κ-CG); stearyl methacrylate (C18); dococyl acrylate (C22); montmorillonite (MMT); xanthan gum (XG); ammonium sulfate (AS); functionalized-boron nitride nanosheets (f-BNNS); acetonitrile-based supramolecular gel (G-Zn-tpy); oxidized alginate (ADA); acrylamide-modified chitosan (AMCS7); oxidized sodium alginate (OSA); α-lipoic acid (LA).

## Data Availability

Not Applicatable.
